# Arginase Is Essential for Survival of Leishmania donovani Promastigotes but Not Intracellular Amastigotes

**DOI:** 10.1128/IAI.00554-16

**Published:** 2016-12-29

**Authors:** Jan M. Boitz, Caslin A. Gilroy, Tamara D. Olenyik, Dustin Paradis, Jasmine Perdeh, Kristie Dearman, Madison J. Davis, Phillip A. Yates, Yuexin Li, Michael K. Riscoe, Buddy Ullman, Sigrid C. Roberts

**Affiliations:** a Department of Biochemistry and Molecular Biology, Oregon Health & Science University, Portland, Oregon, USA; b Pacific University School of Pharmacy, Hillsboro, Oregon, USA; c VA Medical Center, Experimental Chemotherapy Laboratory, Portland, Oregon, USA; d Department of Molecular Microbiology and Immunology, Oregon Health & Science University, Portland, Oregon, USA; Cornell University

**Keywords:** Leishmania, arginase, polyamines

## Abstract

Studies of Leishmania donovani have shown that both ornithine decarboxylase and spermidine synthase, two enzymes of the polyamine biosynthetic pathway, are critical for promastigote proliferation and required for maximum infection in mice. However, the importance of arginase (ARG), the first enzyme of the polyamine pathway in Leishmania, has not been analyzed in L. donovani. To test ARG function in intact parasites, we generated Δ*arg* null mutants in L. donovani and evaluated their ability to proliferate *in vitro* and trigger infections in mice. The Δ*arg* knockout was incapable of growth in the absence of polyamine supplementation, but the auxotrophic phenotype could be bypassed by addition of either millimolar concentrations of ornithine or micromolar concentrations of putrescine or by complementation with either glycosomal or cytosolic versions of ARG. Spermidine supplementation of the medium did not circumvent the polyamine auxotrophy of the Δ*arg* line. Although ARG was found to be essential for ornithine and polyamine synthesis, ornithine decarboxylase appeared to be the rate-limiting enzyme for polyamine production. Mouse infectivity studies revealed that the Δ*arg* lesion reduced parasite burdens in livers by an order of magnitude but had little impact on the numbers of parasites recovered from spleens. Thus, ARG is essential for proliferation of promastigotes but not intracellular amastigotes. Coupled with previous studies, these data support a model in which L. donovani amastigotes readily salvage ornithine and have some access to host spermidine pools, while host putrescine appears to be unavailable for salvage by the parasite.

## INTRODUCTION

Parasites of the genus Leishmania cause a variety of devastating and often fatal diseases in humans and domestic animals. Leishmaniasis ranges from cutaneous ulcerative lesions to fatal visceralizing infections and affects an estimated 12 million people worldwide (1). Among diseases of parasitic origin, visceral leishmaniasis is the second leading cause of mortality in humans worldwide (2). The heteroxenous pathogen lives as the extracellular, flagellated promastigote within its insect vector, the phlebotomine sandfly, and resides as the intracellular, immotile amastigote within the phagolysosomes of infected macrophages and other reticuloendothelial cells of the mammalian host. Due to the absence of effective vaccines, empirical chemotherapies have offered the only avenue of defense for the treatment of leishmaniasis (3–5). Unfortunately, the current arsenal of drugs used to treat leishmaniasis is far from ideal due to the lack of target specificity and emerging drug resistance. Thus, the need to validate new therapeutic targets and to better understand host-parasite interactions that impact these putative targets is acute.

One pathway that has been validated as an antiparasitic drug target in the evolutionarily related pathogen Trypanosoma brucei is that for polyamine biosynthesis (6–9). Polyamines are ubiquitous aliphatic cations that play vital roles in a variety of fundamental cellular processes, including growth, differentiation, and macromolecular synthesis (10–13). In addition to general functions, spermidine is also vital for the modification and activation of eukaryotic initiation factor 5A in parasites, as well as in higher eukaryotes (14–16). Furthermore, in a reaction unique to trypanosomatids, spermidine is conjugated with two glutathione molecules to produce trypanothione, a thiol reductant that serves to maintain the intracellular redox balance and for defense against oxidative stress (17–20). Because of the requirement for polyamines in parasites, inhibitors of polyamine pathway enzymes represent a rational paradigm for the treatment of parasitic diseases (20–25). d,l-α-difluoromethylornithine (DFMO), a suicide inhibitor of ornithine decarboxylase (ODC), the enzyme that converts ornithine to putrescine, has shown remarkable therapeutic efficacy in treating African sleeping sickness caused by *Trypanosoma brucei gambiense* (6–9). DFMO is also effective at killing other genera of protozoan parasites (26–29) and has been found to reduce Leishmania infections in mouse and hamster models (30–32).

The polyamine pathway in Leishmania consists of four enzymes: arginase (ARG), ODC, spermidine synthase (SPDSYN), and *S*-adenosylmethionine decarboxylase (ADOMETDC). ARG, the first and committed step in polyamine biosynthesis, converts arginine to ornithine, which is subsequently metabolized to the diamine putrescine by the catalytic action of ODC. The enzyme SPDSYN then generates spermidine via the addition of an aminopropyl group that is donated from decarboxylated *S*-adenosylmethionine, the product of ADOMETDC. Spermine, a prevalent polyamine of higher eukaryotes, is not made or metabolized further by Leishmania (33), and there is no spermine synthase (SPMSYN) homolog in the leishmanial genome (34). ODC, SPDSYN, and ADOMETDC have all been validated as essential for survival and growth of the promastigote form of L. donovani, as gene knockouts of each enzyme confer polyamine auxotrophy to the parasite and mutants cannot grow in the absence of appropriate polyamine supplementation (33, 35, 36).

Although ARG has not yet been validated as crucial for L. donovani promastigote survival, null mutants at the *ARG* locus have been created in L. mexicana, L. major, and L. amazonensis (37–39), species that are etiologic agents of cutaneous leishmaniasis. These Δ*arg* strains are all auxotrophic for polyamines and exhibit significantly reduced infectivity levels in mice compared to wild-type parasites, although they are all still able to establish infections (38–41). These findings imply that cutaneous amastigotes are able to access ornithine and/or polyamines to some extent from the phagolysosome but demonstrate nevertheless that the parasite ARG is required for maximum infection by these Leishmania species. In contrast, Δ*odc* and Δ*spdsyn* knockouts in L. donovani both elicit striking diminutions in parasite loads in infected mouse organs, although the impact of the Δ*odc* lesion on parasite burdens in both liver and spleen is much greater than that of the Δ*spdsyn* genetic alteration (42, 43). The basis for the observed variations in infection levels among the visceralizing L. donovani Δ*odc* and Δ*spdysn* mutants and the cutaneous Leishmania Δ*arg* species is unknown.

Interestingly, the polyamine pathway of Leishmania is partitioned between the cytosol and the glycosome (37), a peroxisome-like microbody unique to trypanosomatids (44, 45). ARG is located in the glycosome, while ODC, SPDSYN, and ADOMETDC are cytosolic enzymes (37). This discrete cellular segregation of ARG and the downstream polyamine pathway enzymes may afford better control of polyamine biosynthesis and/or facilitate spatial partitioning of the amino acid arginine for polyamine versus protein biosynthesis. Mislocalization of ARG to the cytosol in both L. mexicana and L. amazonensis dramatically reduced parasite burdens, implying that the glycosomal milieu, although not critical for ARG function in promastigotes (37), is indispensable for proper ARG function in amastigotes (39, 46).

To address the differential impacts of Δ*arg* lesions in cutaneous strains on infectivity with those in genes encoding downstream polyamine enzymes in L. donovani and to assess the importance of glycosomal targeting of ARG in both activity and infection, we created and characterized L. donovani Δ*arg* strains. We found that ARG is essential for polyamine biosynthesis *de novo* and for promastigote growth in unsupplemented media. The Δ*arg* deletion triggered a one order of magnitude reduction in parasite burdens in livers of mice after a 4-week standard infection but did not impact parasite loads in spleens. A comparison of murine infectivity in Δ*arg*, Δ*odc*, and Δ*spdsyn* lines supports a model in which L. donovani amastigotes in the phagolysosome scavenge ornithine efficiently and salvage spermidine to some extent, while putrescine acquisition is basically insufficient to support amastigote maintenance.

## RESULTS

### L. donovani ARG.

The coding and flanking sequences for the *ARG* locus of L. donovani (LdBPK_351490.1) were obtained from www.genedb.org. A multisequence alignment showed that the L. donovani ARG open reading frame (ORF) is 97%, 96%, and 95% identical to the predicted ARG proteins of L. major, L. mexicana, and L. amazonensis, respectively (see Fig. S1 in the supplemental material). Like other previously reported Leishmania ARG sequences (37, 39), the L. donovani gene encodes a C-terminal tripeptide that mediates the translocation of the protein to the glycosome. It should be noted that the *ARG* genes from L. donovani and L. major, both Old World Leishmania species, encompass an AKL C-terminal triad, while the *ARG* gene from L. mexicana and L. amazonensis, both New World Leishmania species, encode an SKL C-terminal tripeptide. Both AKL and SKL are archetypical topogenic signals for targeting proteins to the glycosome (47, 48). The L. donovani ARG protein is 43% and 39% identical to the two human ARG enzymes, ARGI and ARGII, respectively.

### Creation and genotypic characterization of Δ*arg* mutants and add-back cell lines.

**FIG 1:**
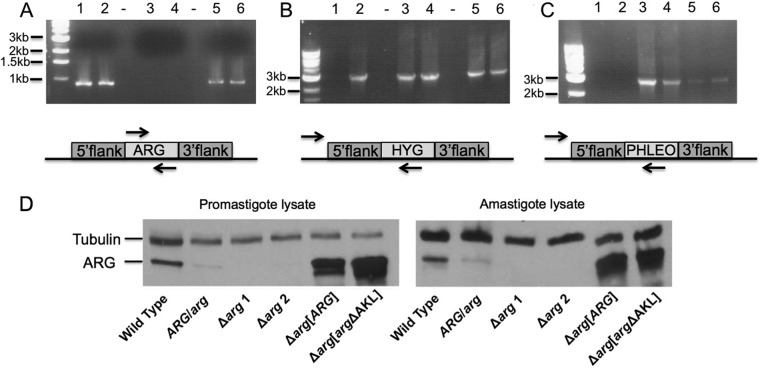
Genotypic and phenotypic analysis of genetically manipulated parasites. (A, B, and C) Genomic DNA from the following parasite strains was used as the template: lanes 1, wild type; lanes 2, *ARG/arg* heterozygote; lanes 3, Δ*arg* mutant 1; lanes 4, Δ*arg* mutant 2; lanes 5, Δ*arg*[*ARG*] mutant; lanes 6, Δ*arg*[*arg*ΔAKL] mutant. (A) Primers designed to encompass the coding region were used to amplify the *ARG* gene. (B) The forward primer sequence was located in a region upstream of the 5′ flanking sequence (upstream of the targeting construct), and the reverse primer sequence was located within the hygromycin resistance gene. (C) The same sense primer as that used for panel B was used, and the antisense primer sequence was located within the phleomycin resistance gene. (D) Western blot analyses were performed with cell extracts prepared from wild-type, *ARG/arg* heterozygote, Δ*arg* mutant 1, Δ*arg* mutant 2, Δ*arg*[*ARG*], and Δ*arg*[*arg*ΔAKL] parasites. Parasites were fractioned by SDS-PAGE and the blot probed with polyclonal antibodies against L. mexicana ARG and a commercial monoclonal antibody that recognizes tubulin. The tubulin antibody was employed to verify equal loading of protein on all lanes.

**FIG 2:**
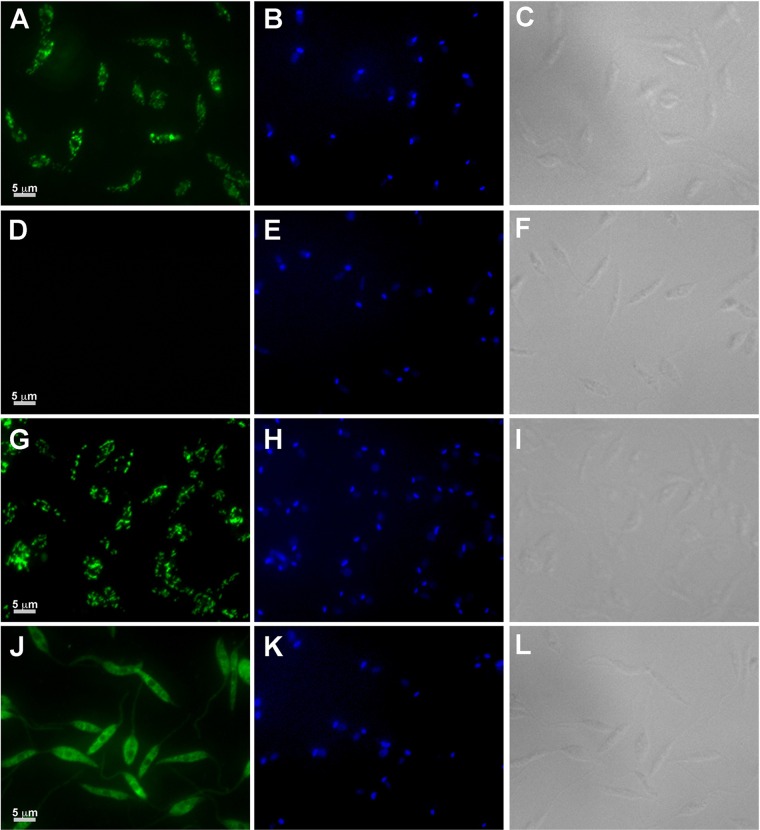
Localization of ARG and argΔAKL. Wild-type (A to C), Δ*arg* (D to F), Δ*arg*[*ARG*] (G to I), or Δ*arg*[*arg*ΔAKL] (J to L) L. donovani promastigotes were subjected to immunofluorescence analysis using rabbit anti-ARG polyclonal antibodies (A, D, G, and J). Goat anti-rabbit Oregon green-conjugated secondary antibody was used to detect the ARG primary antibodies. Parasites were also stained with DAPI (B, E, H, and K) and photographed using differential interference contrast (DIC) (C, F, I, and L).

To investigate the functional role of ARG in parasite growth and infectivity, gene deletion mutants were created by double targeted gene replacement from wild-type L. donovani. *ARG/arg* heterozygotes were first generated by replacing one copy of *ARG* with either a HYG or PHLEO drug resistance marker, and the expected chromosomal rearrangements were confirmed by PCR. Both *ARG/arg* heterozygotes were then subjected to a second round of transfection to generate Δ*arg* null mutants, and two clonal Δ*arg* null mutant cell lines, derived from a HYG heterozygote, were selected for further analysis. In addition, add-back lines were made via insertion of the *ARG* coding region or a mutated *arg* gene lacking the C-terminal AKL glycosomal topogenic signal into the ribosomal locus of a Δ*arg* cell line. Both PCR and Western blot analyses were employed to confirm the predicted genotypes of the mutant parasites (Fig. 1). The PCR analysis demonstrated that the *ARG* coding region could not be detected in the Δ*arg* background but was present in wild-type, heterozygous, and add-back parasites (Fig. 1A). Western blot analysis with lysates harvested from either promastigotes or axenic amastigotes confirmed the absence of ARG protein from Δ*arg* parasites and the presence of ARG protein in wild-type, heterozygous, Δ*arg*[*ARG*], and Δ*arg*[*arg*ΔAKL] cells (Fig. 1D). Somewhat surprisingly, the heterozygous parasites expressed very little ARG protein compared to the much more robust expression of ARG protein in wild-type parasites (Fig. 1D), an observation that could be ascribed to allele-specific expression differences. Immunofluorescence studies confirmed that the wild-type and Δ*arg*[*ARG*] parasites expressed ARG in the glycosomes and that the Δ*arg*[*arg*ΔAKL] cell line produced Arg protein that mislocalized to the cytosol (Fig. 2).

### Nutritional assessment of the L. donovani Δ*arg* mutant.

**FIG 3:**
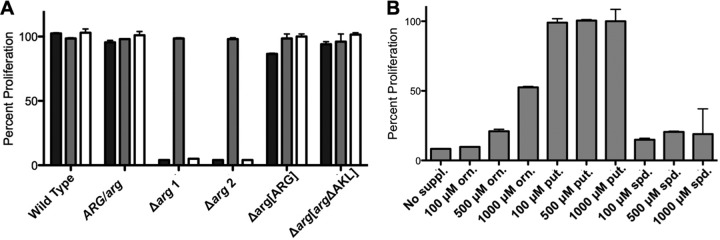
Growth phenotypes of genetically modified promastigotes. (A) Growth phenotypes of wild-type, *ARG/arg*, Δ*arg* mutant 1, Δ*arg* mutant 2, Δ*arg*[*ARG*], and Δ*arg*[*arg*ΔAKL] promastigotes in unsupplemented medium (black bars) or medium supplemented with 100 μM putrescine (gray bars) or 100 μM ornithine (white bars) are shown. (B) Growth phenotypes of the Δ*arg* parasites in unsupplemented medium and medium supplemented with three different concentrations (100 μM, 500 μM, and 1,000 μM) of ornithine (orn.), putrescine (put.), or spermidine (spd.) are depicted. Parasites were incubated at a density of 5 × 10^5^ parasites/ml, and cell viability was evaluated after 5 days by resazurin-to-resorufin conversion. The capacity of wild-type parasites to metabolize resazurin in unsupplemented medium was equated to 100% proliferation. The experiments were set up in duplicate and repeated three times with similar results.

**FIG 4:**
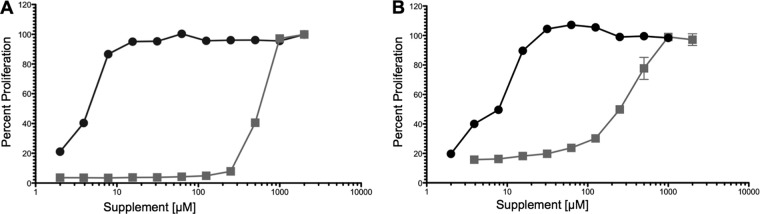
Requirements for ornithine and putrescine in Δ*arg* promastigotes and axenic amastigotes. Δ*arg* promastigotes (A) and Δ*arg* axenic amastigotes (B) were incubated in serial dilutions of putrescine (black circles) or ornithine (gray squares). Supplement concentrations ranged from 1 to 2,000 μM. Promastigotes were seeded at 5 × 10^5^/ml in 96-well plates and incubated in DME-L at 27°C, while axenic amastigotes were seeded at 5 × 10^5^/ml in 96-well plates and incubated in a modified acidic medium at 37°C. After 5 days, the ability of parasites to metabolize resazurin was assessed by fluorescence, and readings obtained with the highest supplement concentrations equated to 100% proliferation. The experiments were set up in duplicate and repeated three times with similar results.

Growth assays confirmed that the Δ*arg* lesion conferred a conditionally lethal growth phenotype to L. donovani. Only the Δ*arg* cell line was unable to grow in the absence of ornithine or polyamine supplementation, while wild-type, Δ*arg*[*ARG*], and Δ*arg*[*arg*ΔAKL] parasites grew at comparable rates and to similar densities in polyamine-free medium (Fig. 3A). The phenotypic consequences of the genetic lesion in Δ*arg* parasites could be avoided by supplementation of the medium with the downstream metabolite putrescine (Fig. 3A and B), while spermidine, the penultimate polyamine in Leishmania, did not rescue Δ*arg* proliferation at concentrations of up to 1,000 μM (Fig. 3B). The concentrations of ornithine and putrescine that restored Δ*arg* cell growth were determined by incubation of the knockouts in medium containing different ornithine or putrescine levels (Fig. 3B and 4). An initial screen indicated that optimal growth of Δ*arg* parasites could be achieved at 100 μM putrescine, whereas supplementation of the medium with 100 μM ornithine had essentially no growth-stimulatory effect (Fig. 3A). Only modest growth could be achieved at 500 μM to 1 mM ornithine (Fig. 3B). Incubation of Δ*arg* promastigotes in medium containing serial dilutions of either ornithine or putrescine revealed effective concentrations of additive that achieved 50% maximum cell densities (EC_50_s) of 750 ± 229 μM for ornithine and 7.5 ± 5 μM for putrescine (Table 1 and Fig. 4A). Because ornithine and putrescine incorporation by the intracellular amastigote form is ultimately key for the clinical validation of ARG as a potential therapeutic target, the requirement for ornithine and putrescine was also assessed in axenic amastigotes. The EC_50_s calculated for ornithine and putrescine were 383 ± 147 μM and 6.25 ± 3.86 μM, respectively (Fig. 4B).

**TABLE 1:** EC_50_s for ornithine and putrescine in L. donovani, L. mexicana, and L. major Δ*arg* mutants

Supplement	EC_50_ (μM) for Δ*arg* mutant of^*a*^:		
	L. donovani	L. mexicana	L. major
Ornithine	750 ± 229	500*	1,000**
Putrescine	7.5 ± 4.95	2*	30**

a EC_50_s for *L. donovani* Δ*arg* promastigotes were determined, as shown in Fig. 4, by incubating parasites in serial dilutions of ornithine or putrescine for 5 days. Proliferation was evaluated by assessing resazurin conversion as a measure of metabolic activity. Each value represents the means and standard deviations from three independent experiments set up in duplicate. The EC_50_s for L. mexicana Δ*arg* and L. major Δ*arg* mutants have been reported elsewhere (*, reference 37; **, reference 38).

**FIG 5:**
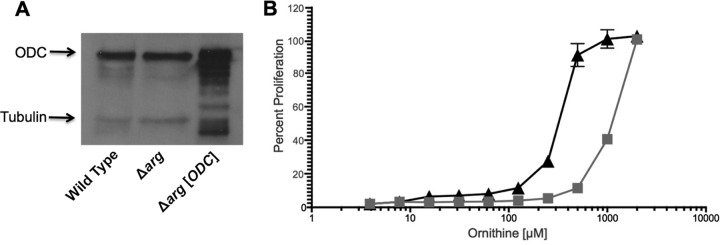
Ornithine requirement for Δ*arg*[*ODC*] promastigotes. (A) Western blot analysis was performed with cell lysates prepared from wild-type parasites, the parental Δ*arg* line, and the Δ*arg*[*ODC*] ODC overproducer strain. Parasite lysates were fractioned by SDS-PAGE and the blot probed with polyclonal antibodies against L. donovani ODC and an anti-tubulin antibody as a loading control. (B) Growth phenotypes of Δ*arg* (gray squares) and Δ*arg*[*ODC*] (black triangles) promastigotes were established in increasing concentrations of ornithine. Parasites were incubated at 5 × 10^5^ parasites/ml, and percent proliferation was evaluated after 5 days via the ability of parasites to convert resazurin to resorufin as assessed by fluorescence, and readings obtained with the highest supplement concentrations were equated with 100% proliferation. The experiments were set up in duplicate and repeated three times with similar results.

Previous studies with L. mexicana promastigotes revealed that transport capacities for putrescine and ornithine are similar and thus cannot explain the difference in ornithine and putrescine concentrations that are required to efficiently bypass a Δ*arg* lesion (37). However, ornithine levels are much higher than those for putrescine in L. mexicana (37), suggesting that ODC is the rate-limiting step in polyamine production. To test this conjecture, we investigated whether overproduction of ODC in the Δ*arg* parasites could reduce ornithine requirements for parasite proliferation by constructing a Δ*arg* derivative line, the Δ*arg*[*ODC*] strain, that overexpressed *ODC*. Overproduction of the ODC protein in the Δ*arg*[*ODC*] line was confirmed by Western blotting (Fig. 5A). Growth analysis in serial dilutions of ornithine revealed that the Δ*arg*[*ODC*] parasites indeed required smaller amounts of ornithine than the parental Δ*arg* parasites (Fig. 5B).

### Effect of ARG inhibitors on promastigote proliferation.

Because ARG is essential for promastigote proliferation, the arginine analogues N^ω^-hydroxy-l-arginine (NOHA), N^ω^-hydroxy-nor-l-arginine (nor-NOHA), *S*-(2-boronoethyl)-l-cysteine (BEC), and 2(*S*)-amino-6-boronohexanoic acid (ABH), all potent inhibitors of the recombinant L. mexicana ARG (49), were tested for their growth-inhibitory effects against L. donovani promastigotes. EC_50_s of 275 μM and 610 μM were calculated for NOHA and nor-NOHA, respectively (data not shown). Addition of putrescine negated the observed growth inhibition caused by NOHA and nor-NOHA completely (EC_50_ of >1,000 μM). That the modest growth inhibition triggered by NOHA and nor-NOHA in L. donovani could be reversed by putrescine supplementation indicates that ARG is the intracellular target of the two ARG enzyme inhibitors. The other two ARG inhibitors tested, ABH and BEC, were less effective growth inhibitors. An EC_50_ of ∼2.5 mM was obtained for ABH, while BEC displayed no growth-inhibitory effect on promastigote growth up to 5 mM (data not shown). Interestingly, NOHA exhibited efficacy against L. major (38) but not against L. mexicana promastigotes (46). The relatively poor efficacy of the ARG enzyme inhibitors, at least for L. mexicana and L. donovani, can be ascribed to potentially poor uptake of the charged compounds (46, 49).

### Infectivity analyses.

**FIG 6:**
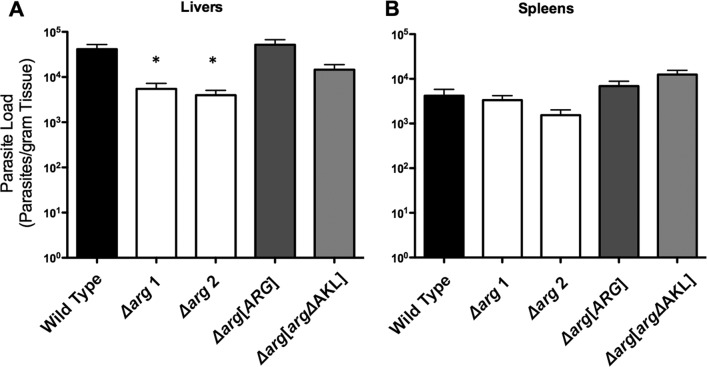
Parasite burden in mice infected with wild-type and genetically modified parasites. Five separate groups of five BALB/c mice were infected with either wild-type, Δ*arg* mutant 1, Δ*arg* mutant 2, Δ*arg*[*ARG*], or Δ*arg*[*arg*ΔAKL] stationary-phase promastigotes via tail vein inoculation. Mice were sacrificed after 4 weeks, and parasite loads in liver (A) or spleen (B) preparations were determined by limiting dilution. Statistical analyses were performed using the paired *t* test and revealed significant differences in liver infectivity between wild-type and Δ*arg* mutant 1 parasites, as well as between the wild type and the Δ*arg* mutant 2 strain (denoted with an asterisk).

To determine the impact of an *ARG* deficiency on infectivity, parasite burdens in both liver and spleen tissues were assessed in BALB/c mice inoculated with either wild-type, Δ*arg*, Δ*arg*[*ARG*], or Δ*arg*[*arg*ΔAKL] parasites. Mice were sacrificed 4 weeks postinfection, since a time course experiment had previously determined that hepatic parasite burdens in our mouse model peaked between two and 4 weeks while splenic parasite loads were much lower but remained at steady levels in the spleen for up to 10 weeks (Fig. S2). Previous infectivity studies with Δ*odc* and Δ*spdsyn* L. donovani strains were also performed 4 weeks postinoculation (42, 43). The average liver parasite loads of the five mice infected with wild-type parasites varied between 10^4^ and 10^5^ parasites/g, and the average parasite numbers of mice infected with the two independent Δ*arg* knockout strains were an order of magnitude lower (Fig. 6A). Statistical analysis (paired *t* test) revealed a significant difference in liver parasite numbers harvested from mice injected with either wild-type or knockout parasites (*P* values of 0.0261 and 0.0279 for a comparison between the wild type and the Δ*arg* mutants 1 and 2). Parasite burdens in livers obtained from mice infected with the Δ*arg*[*ARG*] add-back parasites were equivalent to those of mice inoculated with wild-type parasites, intimating that the infectivity deficit incurred by the Δ*arg* lesion can be ascribed to ARG loss and not to some ancillary genetic alteration that occurred through the extended creation of the null line. Parasite loads in livers of mice infected with Δ*arg*[*arg*ΔAKL] parasites expressing the misolocalized cytosolic *arg* were intermediate between those from mice injected with wild-type and Δ*arg* parasites. Only inconsequential differences in parasite numbers were observed in splenic preparations among mice infected with either wild-type, Δ*arg* knockout, or add-back parasites (Fig. 6B).

## DISCUSSION

To evaluate the functional role of ARG, the first and committed step in polyamine biosynthesis in L. donovani, a Δ*arg* knockout was created by double targeted gene replacement and characterized with respect to its growth and infection capabilities. The Δ*arg* lesion conferred polyamine auxotrophy to the parasite that could be rescued by supplementation of the growth medium with either ornithine or putrescine. The finding that putrescine could fully rescue the deleterious consequences of a Δ*arg* null mutation in both promastigotes and axenic amastigotes authenticates that the sole function of ARG in L. donovani is to support polyamine biosynthesis, a result similar to previous findings obtained with promastigotes of Δ*arg* null mutants of several cutaneous species of Leishmania (37–39). In contrast, addition of spermidine to the culture medium up to a concentration of 1 mM did not enable growth of Δ*arg* promastigotes (Fig. 3B). Because spermidine is efficiently transported into Leishmania (50–52), the observation that spermidine could not rescue the polyamine auxotrophy of the L. donovani Δ*arg* mutant implies that putrescine is an essential metabolite for proliferation of L. donovani promastigotes. Coupled with previous findings that the L. donovani Δ*spdsyn* mutant accumulates putrescine as a result of the genetic block but also display polyamine auxotrophy (35), our data establish for the first time that both of the polyamines found in L. donovani, putrescine and spermidine, are each of their own accord critical for the survival and growth of the parasite. That spermidine by itself is indispensable for parasite proliferation is unsurprising, since the polyamine is a component of two vital downstream reactions. Spermidine is a constituent of trypanothione, the thiol reductant in Leishmania and related trypanosomatids that replaces the role of glutathione in mammalian systems (17–20), and hypusine, an unusual amino acid that is the result of a posttranslational modification in eukaryotic translation initiation factor 5A and is essential for its proper function (14–16). In contrast to spermidine, a specific function for putrescine is unknown other than that the diamine serves as a general aliphatic polycation. Finally, the fact that supplementation of the growth medium with spermidine cannot rescue a Δ*arg* deficiency in L. donovani (Fig. 3B), together with our previous observation that spermine supplementation cannot rescue a Δ*odc* deficiency (33), proves the absence of a back-conversion pathway from spermine to spermidine and putrescine in Leishmania that is operative in mammalian cells (53, 54).

The basis for why higher concentrations of ornithine than putrescine are required to rescue the phenotypic consequences of a Δ*arg* deficiency in both promastigotes and axenic amastigotes is not known. Ornithine and putrescine uptake rates into L. mexicana promastigotes (37) are roughly equivalent, although an analysis of ornithine and putrescine uptake into L. donovani promastigotes has not been undertaken. Because L. mexicana promastigotes contain vastly more ornithine (∼130 nmol/10^7^ parasites) than putrescine (∼2.5 nmol arginine/10^7^ parasites) (37), we speculate that ODC is the rate-limiting enzyme in polyamine biosynthesis, converting only small amounts of stockpiled ornithine into putrescine. Our finding that overexpression of ODC in the Δ*arg* background reduced the levels of ornithine required to enable growth (Fig. 5) supports this conjecture. Little is known about the regulation of polyamine synthesis in trypanosomatids, although it has long been established that mammalian cells utilize an intricate regulatory system to ensure low and stable polyamine levels (55–58). Our supposition that ODC is a rate-limiting enzyme in Leishmania together with the recent discovery of the controlled expression of prozyme, necessary for the activity of ADOMETDC, in African trypanosomes (24, 59–62) suggests that polyamine biosynthesis is also regulated in trypanosomatids.

The cellular localization of ARG does not appear to be crucial for its function in L. donovani promastigotes or axenic amastigotes, as add-back parasites expressing glycosomal or cytosolic mislocalized ARG both exhibit polyamine prototrophy (Fig. 3). Similar results have also been observed in L. mexicana and L. amazonensis promastigotes (37, 39). However, mislocalization of ARG to the cytosol in both L. mexicana and L. amazonensis, unlike L. donovani (Fig. 6), dramatically reduced parasite burdens, implying that the glycosomal milieu, although not critical for ARG function in promastigotes, is indispensable for proper ARG function in amastigotes of the two species (39, 46). Why the cellular environment appears to be important for ARG function in intracellular L. mexicana and L. amazonensis amastigotes but not extracellular parasites is unclear but could be due to limiting availability of arginine in the host cell (63).

There exists an extraordinary positional variation in the impacts that specific lesions in polyamine biosynthesis exert on L. donovani infection capacity. Among the three null L. donovani mutants deficient in polyamine biosynthesis that have been tested in mice, the Δ*odc* deletion effectively obliterates the capacity of the parasite to trigger a mouse infection (42), the Δ*spdsyn* lesion shows reductions of three and two orders of magnitude in parasite burdens in liver and spleen, respectively (43), and the Δ*arg* mutation confers a statistically significant 10-fold reduction in liver parasite loads but does not compromise splenic parasite numbers (Fig. 6). All determinations of parasite loads in these experiments were performed 4 weeks postinfection, the interval in which both hepatic and splenic infections were maximal in our system (see Fig. S2 in the supplemental material). It should be noted, however, that although it has long been known that hepatic infections of visceralizing Leishmania strains are self-limiting in mice, splenic parasite numbers in murine infections tend to increase throughout month-long infections (64–68). The basis for the discrepancies in the time courses of splenic infections between our laboratory and others (64–68) is unclear and was not pursued further, because the objective was to compare the impacts of specific genetic defects in the polyamine pathway upon infection.

**FIG 7:**
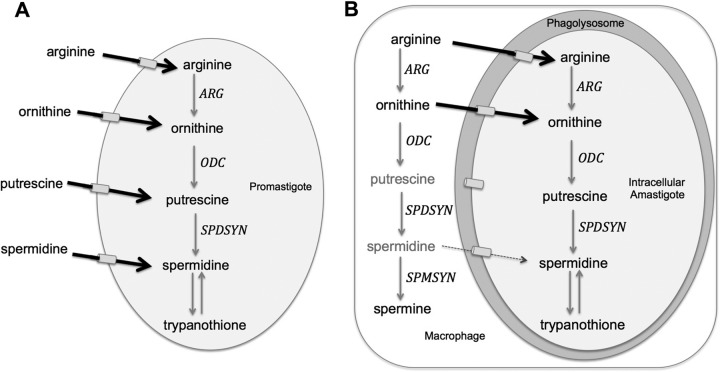
Polyamine salvage model for promastigotes and intracellular amastigotes. (A) L. donovani promastigotes efficiently transport arginine, ornithine, and polyamines. Arginine is an essential amino acid (88, 89), while promastigotes are able to synthesize ornithine and polyamines endogenously. Putrescine and spermidine, but not ornithine, are essential for parasite growth and survival, and gene deletion mutants depend on polyamine supplementation of the media. (B) L. donovani amastigotes reside inside the phagolysosome (shaded gray) of host macrophages. Our data support a model wherein ornithine salvage is hypothesized to be efficient, while spermidine and especially putrescine salvage pools are too limited to support robust infections. Although amastigotes have putrescine and spermidine transporters (50, 51), both polyamines have been shown to be rapidly metabolized to spermine in macrophages (69) and thus are not accessible to the intracellular parasite. Spermine cannot meet the polyamine requirements of Leishmania parasites (33).

Although it is problematic to compare infectivity phenotypes between visceral and cutaneous strains of Leishmania, a similar statistically meaningful reduction in parasite virulence, as determined by footpad lesion size, was also observed with Δ*arg* L. mexicana, L. major, and L. amazonensis mutants, although all three cutaneous Δ*arg* strains retained significant infection capacity (38–41). The growth deficits of the Δ*odc* and Δ*spdsyn* L. donovani mutants cannot be due to a lack of polyamine transport capacity, because axenic amastigotes are able to efficiently import both putrescine and spermidine, the products of ODC and SPDSYN, respectively (50, 51). Proficient putrescine uptake is also functionally demonstrated by the observation that Δ*arg* axenic amastigotes grew well in medium supplemented with micromolar concentrations of putrescine (Fig. 4). Most likely, the divergence in the infection capacities among the Δ*arg*, Δ*odc*, and Δ*spdsyn* L. donovani mutants reflects the relative ornithine and polyamine pools within the phagolysosome of infected macrophages. It is logical to propose that the parasite has access to ample ornithine to bypass a Δ*arg* lesion, essentially no putrescine to evade the consequences of a Δ*odc* deficiency, and intermediate spermidine pools that enable partial bypass of a Δ*spdsyn* mutation. It is intriguing that Δ*arg* axenic amastigotes require much higher concentrations of ornithine than putrescine for optimal growth (Fig. 4B), thus the physiological concentrations of ornithine would have to be high in the phagolysosome to circumvent the *arg* deletion. The model of putrescine and relative spermidine deficiencies in the host cell is supported by the observation that macrophages rapidly convert these smaller polyamines to spermine (69). Previous Leishmania infection models have proposed that an increased host ARGI activity in infected macrophages provides polyamines for salvage and parasite proliferation (70, 71); however, our findings imply a refined model where ornithine, rather than polyamines, is available for salvage by the parasite (Fig. 7). This hypothesis can be tested in the future by measuring nutrient concentrations in macrophages and, ideally, phagolysosomes.

## MATERIALS AND METHODS

### Materials, chemicals, and reagents.

Resazurin, G418, hygromycin, putrescine, and ornithine were purchased from VWR International (Radnor, PA). Phleomycin was procured from Thermo Fisher Scientific (Waltham, MA). N^ω^-hydroxy-l-arginine (NOHA), N^ω^-hydroxy-nor-l-arginine (nor-NOHA), *S*-(2-boronoethyl)-l-cysteine (BEC), and 2(*S*)-amino-6-boronohexanoic acid (ABH) were bought from Enzo (Farmingdale, NY). Restriction enzymes were acquired from New England BioLabs (Ipswich, MA). The Wizard SV gel and PCR clean-up system was purchased from Promega (Madison, WI), and the DNeasy kit was obtained from Qiagen Inc. (Valencia, CA). The pCR 2.1-TOPO vector and synthetic oligonucleotides were acquired from Invitrogen Corp. (Carlsbad, CA), and the Advantage HF2 DNA polymerase mix was purchased from BD Bioscience (Palo Alto, CA). The pRP-M vector was a gift from Phillip A. Yates, Oregon Health & Science University.

### Cell lines, cell culture, and assessment of growth phenotypes.

All genetically manipulated parasites were derived from the wild-type LdBob strain of L. donovani (72) that was originally obtained from Stephen M. Beverley (Washington University, St. Louis, MO). Wild-type LdBob and genetically manipulated derivative strains were routinely cycled between the promastigote and axenic amastigote stages at 26°C, pH 7.4, and 37°C, pH 5.5, respectively, using previously reported cell culture conditions, in order to maintain infectivity (73, 74). Promastigotes were incubated in completely defined DME-L (75), while amastigotes were grown in an M199-based medium (73). Wild-type, Δ*arg*[*ARG*], and Δ*arg*[*arg*ΔAKL] parasites were routinely cultured in medium with no ornithine or putrescine supplementation, while the Δ*arg* cells were maintained in medium supplemented with 50 μg/ml hygromycin, 50 μg/ml phleomycin, and 100 μM putrescine.

Growth phenotypes were established by incubating wild-type and mutant L. donovani promastigotes or axenic amastigotes seeded in a volume of 100 μl in 96-well plates at a density of 5 × 10^5^/ml in the absence or presence of various concentrations of ornithine, putrescine, or spermidine as specified. Growth experiments with spermidine added to the medium necessitated replacement of fetal bovine serum with chicken serum to avoid polyamine oxidase-mediated toxicity (35, 76). After 5 days, 10 μl of 250 μM resazurin was added to each well to assess cell density, and plates were incubated for an additional 4 h. Conversion of resazurin to resorufin was evaluated on a BioTek Synergy plate reader by monitoring fluorescence (excitation wavelength, 579 nm; emission wavelength, 584 nm). Graphs were prepared using GraphPad Prism version 4.0 or 6.0f for Mac (GraphPad Software, La Jolla, CA).

### Creation of Δ*arg* parasites.

The Δ*arg* knockouts were generated in the LdBob background by double targeted gene replacement (77, 78). To construct drug resistance cassettes for the replacement of *ARG*, the 5′ *ARG* flanking region was amplified by PCR using the following primers containing HindIII and SalI restriction sites (underlined): forward primer, GCATAAGCTTGATCCCTCAAGCACATTGTGAAG; reverse primer, GCATGTCGACTGACCCTGTCACCACCAGAC. The resulting PCR product was first subcloned into the pCR 2.1-TOPO vector, excised with HindIII and SalI endonucleases, and inserted into the HindIII and SalI sites of pX63-HYG and pX63-PHLEO vectors to generate pX63-HYG-5′F and pX63-PHLEO-5′F, respectively. The 3′ flanking region was then amplified by PCR using the following primers containing SmaI and BglII sites (underlined): forward primer, GGGCCCGCGCATGTGTTCCTTCCAAG; reverse primer, GCATAGATCTGGGAGATGTCAGAGGAGAGGAGAG. The PCR product was first ligated into the pCR 2.1-TOPO vector and then inserted into the SmaI/BglII site of pX63-HYG-5′F to generate pX63-HYG-Δ*arg.* Because of the presence of an SmaI site within the PHLEO coding region of pX63-PHLEO, a different cloning strategy was employed to insert the 3′-flanking region of *ARG* into pX63-PHLEO-5′F. The 3′-flanking region was amplified by PCR using a forward primer containing the BamHI restriction site, GCATGGATCCGCGCATGTGTTCCTTCCAAG, and the above-noted reverse primer containing the BglII site. The resulting PCR product was subcloned into the pCR 2.1-TOPO vector and then introduced into the BamHI/BglII sites of pX63-PHLEO-5′F to generate pX63-PHLEO-Δ*arg*. The correct orientations of the 5′- and 3′-flanking regions within the gene-targeting plasmids were verified by limited nucleotide sequencing of the insert junctions.

The plasmids pX63-HYG-Δ*arg* and pX63-PHLEO-Δ*arg* were digested with HindIIII and BglII to liberate 6-kb and 5.5-kb linear fragments, designated X63-HYG-Δ*arg* and X63-PHLEO-Δ*arg*, respectively. X63-HYG-Δ*arg* and X63-PHLEO-Δ*arg* were purified from DNA agarose gels using the Wizard SV gel and PCR clean-up system according to the manufacturer's protocol and then transfected into parasites using standard electroporation conditions (79). Two heterozygous cell lines, *ARG*/Δ*arg*::HYG and *ARG*/Δ*arg*::PHLEO, were generated first and selected in semisolid agar plates containing 50 μg/ml hygromycin or 50 μg/ml phleomycin, respectively. The genotypes of the two heterozygous cell lines were confirmed by PCR and then subjected to a second round of transfection. The *ARG/*Δ*arg*::HYG heterozygotes were transfected with the PHLEO-Δ*arg* fragment, and the *ARG/*Δ*arg*::PHLEO heterozygotes were transfected with the X63-HYG-Δ*arg* fragment. Potential homozygous Δ*arg*::HYG*/*Δ*arg*::PHLEO (Δ*arg*) knockouts were selected in semisolid agar plates containing 50 μg/ml hygromycin, 50 μg/ml phleomycin, and 200 μM putrescine. Several clones were picked and parasite cultures expanded. The homologous gene replacements were confirmed by PCR (Fig. 1). Two Δ*arg* knockout clones, derived from the hygromycin-resistant heterozygotes (here designated *ARG/arg*), were picked for further analysis.

### Complementation of Δ*arg* cell lines.

To generate a stably complemented add-back line, *ARG* was introduced into Δ*arg* parasites by homologous recombination at the ribosomal locus (80). Two different constructs were generated, one containing the wild-type *ARG* sequence (Δ*arg*[*ARG*]) and one containing a mutated version lacking the C-terminal AKL tripeptide (Δ*arg*[*arg*ΔAKL]), an archetypical signal that targets proteins for glycosomal localization in Leishmania (47, 48, 81). The *ARG* coding region was amplified with the following primers, each containing an SfiI restriction site, GGCCNNNNNGGCC (underlined): forward primer GAGGCCACCTGGGCCTCATCATGGAGCACGTGCAG and reverse primer GAGGCCAGCCCGGCCCGCACACACACATCTACAGTTTGG. A mutated version of the *ARG* coding region was constructed by using a reverse primer where the nucleotides encoding the C-terminal AKL triad were replaced with a stop codon. The following reverse primer was used, with the SfiI site underlined and the stop codon in boldface: GAGGCCAGCCCGGCC**CTA**GCTCTTACGTGGGGTGTAAAGAAGTG. The SfiI fragments were subcloned into pRP-M (80), yielding pRP-M-*ARG* (pRP-*ARG*) and pRP-M-*arg*ΔAKL (pRP-*arg*ΔAKL). The plasmids were linearized by digestion with AvrII and purified with the Wizard SV gel and PCR clean-up system (Promega) according to the manufacturer's protocol. Δ*arg* parasites were transfected with these constructs using standard electroporation conditions (79), and integrated constructs were selected in bulk culture in 20 μg/ml G418. Clonal isolates from the bulk cultures were obtained by plating on semisolid agar plates. Individual colonies were picked and expanded in medium containing 20 μg/ml G418. Genotypes and phenotypes of the Δ*arg*[*ARG*] and Δ*arg*[*arg*ΔAKL] parasites were verified by PCR, Western blot analysis, and localization studies.

### ODC overproducers in the Δ*arg* background.

The construction of the *ODC* overexpression vector pSNBR[*ODC*] has been described previously (82). The Δ*arg*[*ODC*] parasites were generated by transfecting Δ*arg* parasites with the pSNBR[*ODC*] plasmid using standard electroporation conditions (79). Parasites harboring the episomal construct were selected with 100 μg/ml G418 in bulk culture and the correct phenotype verified by Western blot analysis.

### PCR and Western blotting.

Genomic DNA from the wild type, *ARG/arg* heterozygotes, two independent Δ*arg* clones, and the Δ*arg*[*ARG*] and Δ*arg*[*arg*ΔAKL] add-backs was prepared for Southern blot analysis using the DNeasy kit (Qiagen Inc., Valencia, CA) according to the manufacturer's protocol. The following primers were utilized to amplify a 900-bp *ARG* coding region fragment: ATGGAGCACGTGCAGCAG and CACGTGCGATTCTGTAGCG. Additional primers were designed to detect the replacement of the *ARG* gene with either the PHLEO or HYG drug resistance cassette. The forward primer (CCATCGTGCACGTCTATGAG) was designed from sequence upstream of the deletion construct, and reverse primers were selected from the PHLEO (GAAGTCGTCCTCCACGAAGT) or HYG (CCCGCAGGACATATCCAC) coding region. The presence and size of the PCR products were determined on ethidium bromide-stained agarose gels.

Cell lysates from wild-type, *ARG/arg*, Δ*arg*, Δ*arg*[*ARG*], and Δ*arg*[*arg*ΔAKL] cells were prepared from both promastigotes and axenic amastigotes that were in the late exponential growth phase. Lysates were fractionated by SDS-PAGE (83) and blotted onto Immun-Blot polyvinylidene difluoride (PVDF) membranes (Bio-Rad, Hercules, CA), and Western blot analysis was performed according to standard procedures (84). The membranes were probed with polyclonal antibodies raised in rabbits against the purified recombinant L. mexicana ARG protein (46) and commercially available anti-α-tubulin mouse monoclonal antibody (Calbiochem, La Jolla, CA). Cell lysates were also prepared from Δ*arg*[*ODC*] promastigotes and Western blots probed with polyclonal antibodies raised against the purified recombinant L. donovani ODC protein (33).

### Immunofluorescence microscopy.

The immunofluorescence assay was performed on L. donovani promastigotes as described previously (81, 85, 86) using a 1:500 dilution of anti-LdARG antibody and a 1:10,000 dilution of goat anti-rabbit Oregon green-conjugated secondary antibody (Thermo Fisher Scientific, Waltham, MA). Cells were mounted with VECTASHIELD antifade mounting medium with 4′,6-diamidino-2-phenylindole (DAPI) (Vector Laboratories, Burlingame, CA) and photographed on a Zeiss Axiovert 200 inverted microscope (Carl Zeiss Microimaging) using a 63× oil immersion lens. Photos were taken with a Zeiss AxioCam MR camera using AxioVision 4.2 software (Carl Zeiss Microimaging) and compiled using Adobe Photoshop Creative Suite 4.

### Inhibitor studies.

Wild-type promastigotes were incubated in serial dilutions of NOHA, nor-NOHA, ABH, or BEC in the presence or absence of 100 μM putrescine. Parasites were seeded at a density of 3 × 10^5^/ml in a volume of 100 μl in 96-well plates. After 5 days, parasite viability was evaluated by their ability to metabolize resazurin as described above.

### Murine infection studies.

Cohorts of five 6- to 7-week-old female BALB/c mice (Charles River Laboratories, Wilmington, MA) were inoculated via the lateral tail vein with 5 × 10^6^ stationary-phase promastigotes of either the wild type, two independently derived Δ*arg* clones, or the Δ*arg*[*ARG*] or Δ*arg*[*arg*ΔAKL] add-back. Prior to inoculation, all parasite lines were cycled between the promastigote and axenic amastigote stages. Livers and spleens were harvested 4 weeks after injection as described previously (42, 87). Single-cell suspensions from mouse organs were prepared by passage through a 70-μm cell strainer (BD Falcon), and parasite burdens were determined in 96-well microtiter plates using a standardized limiting-dilution assay (42, 87).

## Supplementary Material

Supplemental material
