# Supplementary material for: Arginase Is Essential for Survival of Leishmania donovani Promastigotes but Not Intracellular Amastigotes
Source: Infect Immun. 2016 Dec 29;85(1):e00554-16. doi: 10.1128/IAI.00554-16 (PMC5203656; doi:10.1128/IAI.00554-16)
Supplement: Supplemental material [file supp_85_1_e00554-16__index.html]

Supplemental material 

# Arginase Is Essential for Survival of Leishmania donovani Promastigotes but Not Intracellular Amastigotes

## Supplemental material

- Supplemental file 1 -

  Fig. S1. Multisequence alignment of phylogenetically diverse ARG proteins. Fig. S2. Parasite burden in mice infected with wild-type parasites.

  PDF, 626K
