# Supplementary material for: Arginase Is Essential for Survival of Leishmania donovani Promastigotes but Not Intracellular Amastigotes
Source: Infect Immun. 2016 Dec 29;85(1):e00554-16. doi: 10.1128/IAI.00554-16 (PMC5203656; doi:10.1128/IAI.00554-16)
Supplement: Supplemental material [file IAI.00554-16_zii999091928s1.pdf]

```
H.sapiens ARG1     -------MSAKSRTIGIIGAPFSKGQPRGGVEEGPTVLRKAGLLEKLKEQ------ECDVKDYGDLPFADIPNDSPFQIVKNPR
L.donovani ARG     MEHVQQYKFYKEKKMSIVLAPFSGGQPHSGVELGPDYLLKQGLQQDMEKLGWNTRLERVFDGKAVEARKASDNGDRIGRVKRPR
L.mexicana ARG     MEHVQQYKFYKEKKMSIVLAPFSGGQPHSGVELGPDYLLKQGLQQDMEKLGWDTRLERVFDGKVVEARKASDNGDRIGRVKRPR
L.major ARG        MEHVQQYKFYKEKKMSIVLAPFSGGQPHGGVELGPDYLLKQGLQQDMEKLGWNTRLERVFDGKAVEARKANDNGDRIGRVKRPR
L.amazonensis ARG MEHVQQYKFYKEKKMSIVLAPFSGGQPHSGVELGPDYLLKQGLQQDMEKLGWDTRLERVFDGKVVEARKASDNGDRIGRVKRPR
                             *.:.:.*: **** ***:.*** **  * * ** :.:::       * . ..    .     *.. :  **.**

H.sapiens ARG1     SVGKASEQLAGKVAEVKKNGRISLVLGGDHSLAIGSISGHARVHPDLGVIWVDAHTDINTPLTTTSGNLHGQPVSFLLKELKGK
L.donovani ARG     LTAECTEKIYKCVRRVAEQGRFPLTIGGDHSIALGTVAGVLTVYPDAGVIWVDAHADINTMSGTVSGNLHGCPLSILLGLDRKN
L.mexicana ARG     LTAECTEKIYKCVRRVAEQGRFPLTIGGDHSIALGTVAGVLSVHPDAGVIWVDAHADINTMSGTVSGNLHGCPLSILLGLDREN
L.major ARG        LTAECTEKIYKCVRRVAEQGRFPLTIGGDHSIALGTVAGVLSVYPDAGVIWVDAHADINTMSGTVSGNLHGCPLSILLGLDREN
L.amazonensis ARG LTAECTEKIYKCVRRVAEQGRFPLTIGGDHSIALGTVAGVLSVHPDAGVIWVDAHADINTMSGTVSGNLHGCPLSILLGLDREN
                    ..:.:*::   * .* ::**:.*.:*****:*:*:::*   *:** ********:****   *.****** *:*:**   : :

H.sapiens  ARG     IPDVPGFSWVTPCISAKDIVYIGLRDVDPGEHYILKTLGIKYFSMTEVDRLGIGKVMEETLSYLLGRKKRPIHLSFDVDGLDPS
L.donovani ARG     IP--ECFSWVPQVLKPSKIAYIGLRAVDEEEKKILHDLNIAAFSMHHVDRYGIDKVVSMAIEAISPKGTEPVMVSYDIDTIDPL
L.mexicana ARG     IP--ECFSWVPQVLKPNKIAYIGLRAVDDEEKKILHDLNIAAFSMHHVDRYGIDKVVSMAIEAVSPKGTEPVMVSYDVDTIDPL
L.major ARG        IP--ECFSWVPQVLKPNKIAYIGLRAVDEEEKKILHDLNIAAFSMHHVDRYGINKVVSMAIEAISPKGTEPVMVSYDVDTIDPL
L.amazonensis ARG IP--ECFSWVPQVLKPNKIAYIGLRAVDDEEKKILHDLNIAAFSMHHVDRYGIDKVVSMAIEAVSPKGTEPVMVSYDVDTIDPL
                   **    ****. :.... *.***** **  *: **: *.*   *** .*** **.**:. ::. :  : ..*: :*:*:* :**

H.sapiens ARG1     FTPATGTPVVGGLTYREGLYITEEIYKTGLLSGLDIMEVNPSLGKTPEEVTRTVNTAVAITLACFGLAREGNHKPIDYLNPPK
L.donovani ARG     YVPATGTPVRGGLSFREALFLCERIAECGRLVALDVVECNPLLAATESHVKDTISVGCAIARCMMGETLLYTPRKSAKL----
L.mexicana ARG     YVPATGTPVRGGLSFREALFLCERIAECGRLVALDVVECNPLLAATESHVNDTISVGCAIARCMMGETLLYTPHTSSKL----
L.major ARG        YVPATGTPVRGGLSFREALFLCELIAECGRLVALDVVECNPLLAATESHVKDTISVGCAIARCMMGETLLYTPRKDAKL----
L.amazonensis ARG YVPATGTPVRGGLSFREALFLCERIAECGRLVALDVVECNPLLAATESHVNDTISDGRAIARCMMGETLLYTPHTSSKL----
                   :.******* ***::**.*:: * * : * * .**::* ** *. * ..*. *:. . **: . :* :   . :    *
```

Supplemental FIG. 1: Multisequence alignment of phylogenetically diverse ARG proteins. ARG amino acid sequences from humans and several *Leishmania* species were aligned. Fully conserved amino acids are denoted with a star (*), strongly conserved residues with a colon (:), and weekly conserved residues with a period (.). The terminal tri-peptide signals for glycosomal localization (SKL or AKL) are underlined. Residues important for manganese binding and interactions with arginine are bolded (Ash 2000).

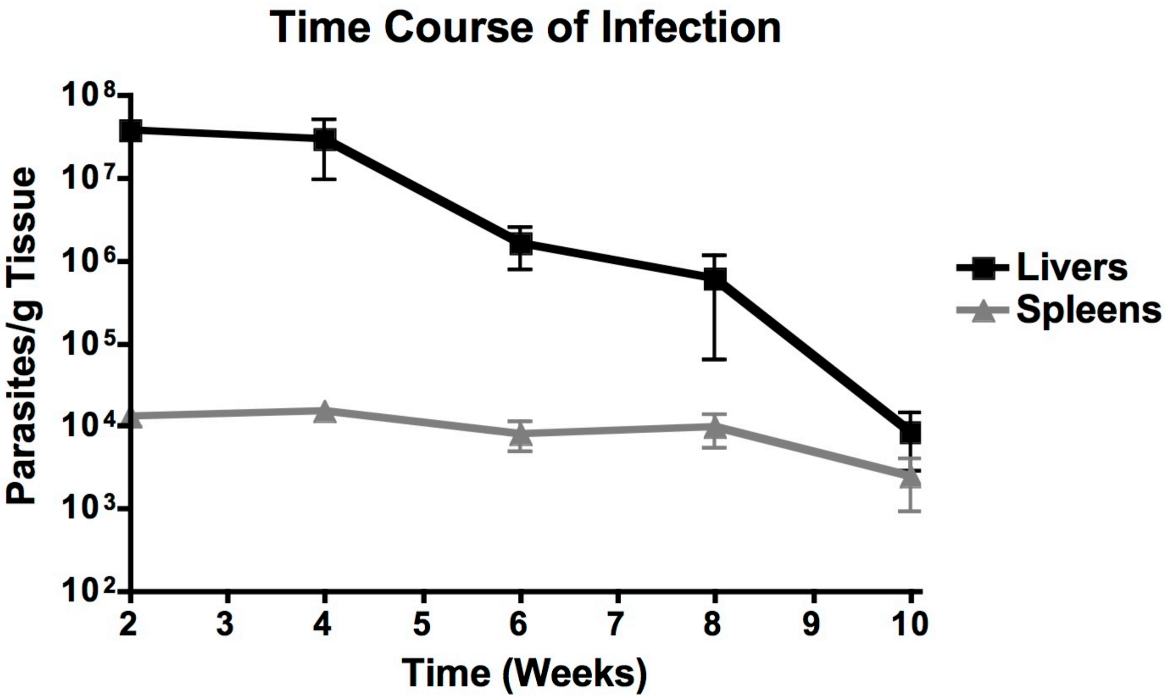

Supplemental FIG. 2: Parasite burden in mice infected with wild type parasites. Ten separate groups of five BALB/c mice were infected with either wild type stationary phase promastigotes via tail vein inoculation. Mice were sacrificed after two, four, six, eight, and 10 weeks and parasite loads in liver (black squares) or spleen (gray triangles) preparations were determined by limiting dilution.
